# A broad spectrum of host plant responses to the actions of the gall midge: case study of *Robinia pseudoacacia* L. and *Obolodiplosis robiniae* (Haldeman)

**DOI:** 10.1186/s12870-022-03914-0

**Published:** 2023-01-10

**Authors:** Aleksandra M. Staszak, Ewelina Ratajczak, Joanna Leśniewska, Alicja Piotrowska-Niczyporuk, Agata Kostro-Ambroziak

**Affiliations:** 1 Laboratory of Plant Physiology, Department of Plant Biology and Ecology, Faculty of Biology, University of Bialystok, Ciołkowskiego 1J, 15-245 Białystok, Poland; 2 Institute of Dendrology, Polish Academy of Sciences, Parkowa 5, 62-035 Kórnik, Poland; 3 Laboratory of Plant Biochemistry, Department of Plant Biology and Ecology, Faculty of Biology, University of Bialystok, Ciołkowskiego 1J, 15-245 Białystok, Poland; 4 Laboratory of Insects Evolutionary Biology and Ecology, Department of Genetic and Zoology, Faculty of Biology, University of Bialystok, Ciołkowskiego 1J, 15-245 Białystok, Poland

**Keywords:** Leaf roll gall, Anatomy, Oxidative, Biotic, Stress, Thiol, Systemic answer

## Abstract

This study aims to provide insights into plant-insect interaction during the formation and development of open gall structure on the leaves of *Robinia pseudoacacia* during gall formation by *Obolodiplosis robiniae*. This was the first time such far-reaching studies were performed at a biochemical and anatomical level. The gall wall is created from a few thick cells covered with epidermis. This parenchymatous nutritive tissue is rich in starch. Sclerenchyma only occurs around the vascular bundles as a result of the lignification of the parenchyma of the bundle sheaths. The level of reactive oxygen species (ROS) in the new structure was reduced and catalase activity was inhibited, which suggests another pathway of ROS decomposition – e.g. by ascorbate or glutathione peroxidase. The gall structure was combined with an increasing level of protein and non-protein thiols. Phenols seems to be a good protective factor; whose level was lower in infected leaflets. Levels of MUFA (monosaturated fatty acids) and SFA (saturated fatty acids) rose, probably as source of food for insects. The amount of fatty acid is positively correlated with the plant response. We detected that non infected leaflets produced C6:0 (hexanoic acid) and C8:0 (octanoic acid) fatty acids connected with odor. Changes in gall color as they develop are connected with photosynthetic pigments degradation (mainly chlorophylls) where the pathway of astaxanthin transformation to fatty acid is considered to be the most important process during gall maturation. Nutritive tissue is composed mainly of octadecanoic acid (C18:0) – a main source of food for *O. robiniae*.

## Introduction

Plants and insect interactions cover a broad range of ecological, structural and biochemical changes. In some cases a gall structure develops, which provides shelter and a source of food for growing larvae (for review see e.g. [1, 2]). An interesting question during gall formation is whether or not the host plants develop some prevention mechanism before global infection. Phenols and tannins contents may play a role in the mechanism against infection [3] but may also be treated as protection for gall inducers [4]. Moreover during lignin biosynthesis, reactive oxygen species (ROS) and phenolic compounds are consumed [5]. The metabolic pathway may be modified by a gall inducer that causes the formation of nutritive tissue with food reserves for insect growth [6]. Fatty acids (FAs) are the major components of lipids in plants [7, 8] and are critical in interactions between plants and insects. For example, insects acquire some FAs from plants to sustain growth, development, and help mate recognition. Studies confirmed this assumption, because many lepidopterans obtain linoleic (C18:2n6) or *α*-linolenic (C18:3n3) acids from plant hosts to sustain their growth [6]. Moreover, plants activate defense responses to insect herbivore attack through the accumulation of fatty acid derivatives such as jasmoniates (e.g. jasmonic acid, JA) which are biosynthesized from C18:3 [8]. The levels of free fatty acids, which are not esterified (to glycerolipids and phospholipids) or not attached to other molecules, are very low in healthy and intact plant tissues. On the other hand, the accumulation of free fatty acids in plants have been shown to increase in response to wounding [9] and insect attack [10]. In particular, MUFAs (monounsaturated FAs) and PUFAs (polyunsaturated FAs) are extensively involved in plant defenses against various biotic stresses via multiple mechanisms [7]. In addition to the important function of fatty acids, some studies indicated that changes in photosynthesis and modification in sugar contents occur during gall formation [11]. The content of chlorophylls may turn out to be a good biochemical indicator of plant health due to their direct role in the photosynthesis. Moreover, the leaf chlorophyll concentration responds, among others agents, to pest presence [12, 13].

The black locust, *Robinia pseudoacacia* L. (Fabaceae), a deciduous tree native to the United States, is nowadays widespread globally, including all continents with warm regions and is among the most widely planted trees in the world [14]. To Europe it was introduced as ornamental plant at the beginning of the seventeenth century but soon it was also cultivated for agricultural uses (for review see [15]) and became the most economic important non-native plant, especially in central Europe [14, 16]. On the other hand, due to its biological plasticity – e.g., high adaptability to environmental stresses, easy propagation (e.g. [16]) – and the fact that for long time it lacked natural enemies in its new habitat, *R. pseudoacacia* has spread out of control. It has been indicated as the thirteenth out of the “100 worst” alien species [17] and one of the 26 alien plants that cause the greatest environmental and socioeconomic impact in Europe [18]. *R. pseudoacacia* affects numerous ecosystem components leading to the modification of plant and lichen communities, decreasing the richness and diversity of soil invertebrate fauna [19–21], to the detriment of birds too [22]. Natural enemies may significantly influence the spread of non-native animals and plants, changing the strength of interactions between these species and the native community – e.g., by decreasing the density and competition of non-native/alien species [23–25]. In its native area of North America, more than 70 insect species prey on *R. pseudoacacia* and some of them were introduced to Europe incidentally [26, 27]. An interesting example of these insects is the gall midge *Obolodiplosis robiniae* (Haldeman, 1847) (Diptera: Cecidomyiidae). It was recorded beyond its native area in 2002 in Japan and South Korea [28, 29], in the following year in Italy [30] and it spread very rapidly through Asia and Europe [31–33]. The larva of *O. robiniae* is monophagous on the genus *Robinia* (primarily *R. pseudoacacia*) developing in the longitudinal rolling gall, along the edge (margin) of the leaflets. Gall protects larvae (1–10 larvae per gall) and provides them with nutrition [32, 34]. Depending on the climatic conditions, *O. robiniae* produces various number of generations but it is considered multivoltine: it can produce from 2 to 3 generations per year in Japan [28], 3–4 generations in Europe [32, 35, 36] and 4–6 generations in China [33]. *O. robiniae* colonizes *R. pseudoacacia* trees of different ages and states [35] and 100% of trees could be affected [32, 37]. *O. robiniae* galls are found on 32–76% of compound leaves and on 3–14% of single leaflets [38]. The number of galls on a single leaflet also differs and could be up to 4–5, and may be formed either by the same or different generations of *O. robiniae* [32].

Unfortunately, there is still gap in our understanding of how *O. robiniae* impacts the host species *R. pseudoacacia*. In the context of *R. pseudoacacia’s* propensity to spread across Europe [39], the question about the impact of natural enemies on the vitality of this plants is very valuable and requires an interdisciplinary approach. Little is known about open structure galls and plant-pathogen interactions. Thus, the aim of our work was to understand if a systemic answer may be found in *O. robiniae* gall formation. We analyzed data from anatomical and different biochemical level to address this question.

## Materials and methods

### Field study

The studied material was collected from single *R. pseudoacacia* trees from May to July of 2019 and 2020 in Białystok (north-eastern Poland, around the campus of University of Bialystok). This tree produces compound leaves (CL) with leaflets that are fully developed in half of May. The CL were collected up to a height of 2 m to represent several variants: two general control GC (1) – leaves without any other symptoms of insect infections. For leaves where one or more leaflet were infected by gall, we collected leaflets without gall and this variant was called internal control – IC (2). Leaflets with gall were LwG (3), and we additionally tested gall as GI (4) and leaflets after gall isolation – LI (5).

**Fig. 1:**
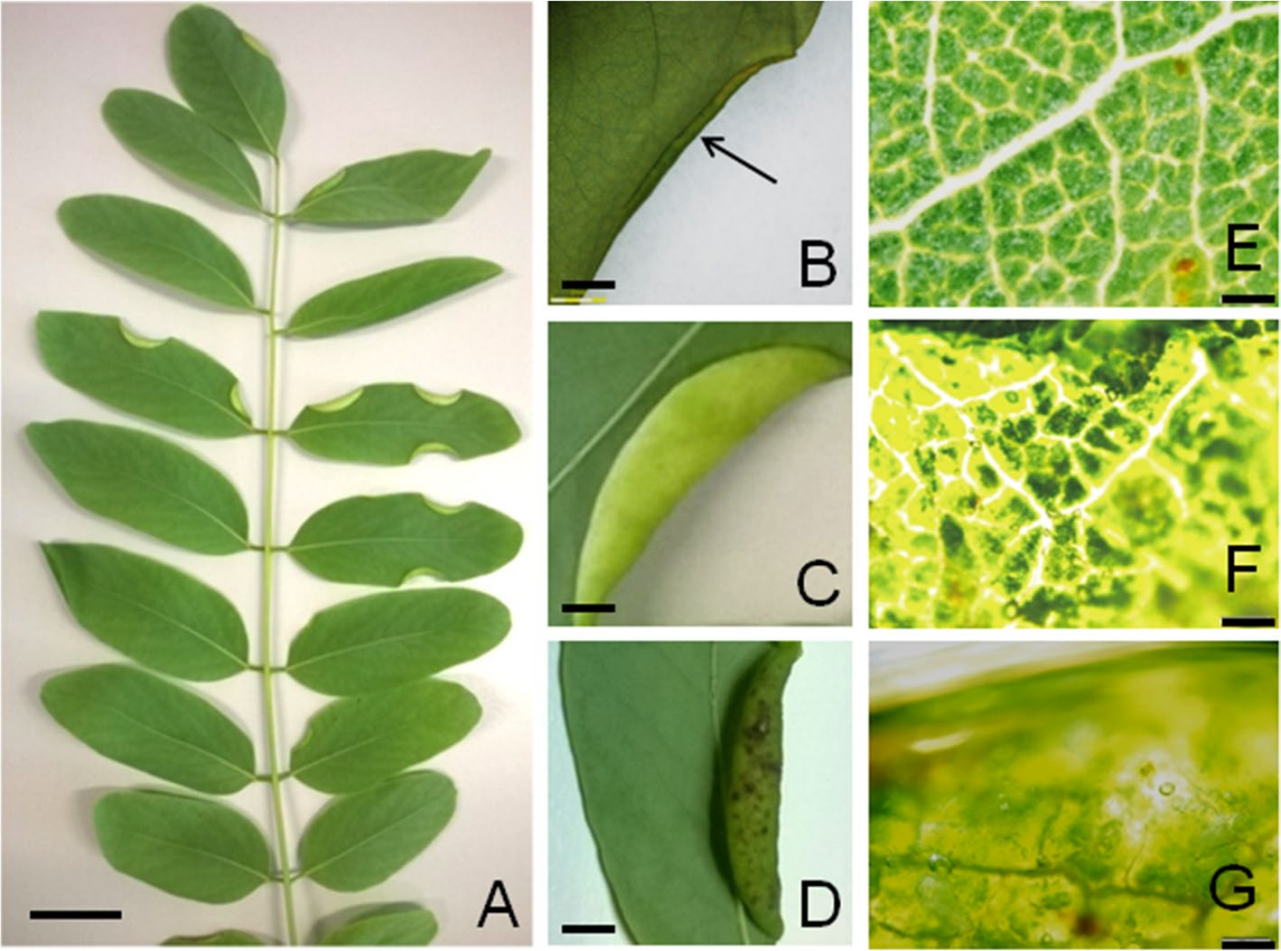
Macroscopic views of galls induced by *O. robiniae* on the *R. pseudoacacia* leaves. (**a**, Bar = 1 cM) The compound leaf of *R. pseudoacacia* with galls on the leaflets (margins of leaflets are rolled downwards). Bar = 1 cm. (**b**-**d**, Bar = 2 mM) Enlarged images of galls; (**b**) The gall initiated on the margin of the leaf blade (arrow); (**c**) Mature, yellowish, spindle-shaped gall; (**d**) Ageing gall, with dark discolorations. **e**-**g** Magnified fragment of the leaf forming the gall wall; fresh material. Bar =100 μM. **e** Green leaf blade before gall formation, view from the upper side (control). **f** Yellow-green, external wall of the mature gall, as in **c**. **g** External wall of the ageing gall, as in **d**

For our study we examined three morphological characteristic stages of gall formation: young, when the gall is small and is similar in color to the rest of the leaf – LwG-Y (Fig. 1b); mature, when the gall turns bright green or yellow – LwG-M (Fig. 1c, *O. robiniae* transfer from 3rd stage of larva to pupa), and senescence, where no larvae were detected inside with signs of aging – LwG-A (Fig. 1d). During the process of aging, the galls gradually dry up. The study protocol respects institutional, national, and international guidelines and legislation related to obtaining material from the field.

### Laboratory studies

The measurements of hydrogen peroxide were done using fresh tissue of each variants. Fresh and frozen (− 80 °C) materials for further biochemical determinations of the contents of proteins, fatty acid, monosaccharides, and photosynthetic pigments during gall formation were also collected. For oxidative parameters and thiols only the gall in medium phase of development were taken. All analyses were done in laboratory conditions till 2 hours after leaves collection.

### Histological studies

For histological studies fresh, frozen and fixed materials of leaflets of *R. psudoacacia* were used. During studies, 4 types of samples were used: control blade of leaflet without gall and gall at three studied stages (young, matured and senescence. Each morphological stages of gall formation have been tested at least on 10 different leaves. Material for histochemistry analysis was fixed chemically in FAA (formaldehyde, glacial acetic acid, ethyl alcohol) for 24 hours, initially under reduced pressure, at room temperature. The material after fixation was dehydrated in 50 and 70% ethanol [40].

The samples were cut *trans*-versely with a sharp razor blade into smaller segments and then into 10 μm sections on cryostat (Cryotome FSE, Thermo Scientific). At the same time handmade scraps were done by razor blade and viewed in water. Histological sections were stained with: Ehrlich haematoxylin, Lugol’s solution (starch detection), and Sudan III (lipid detection) [40, 41]. Larger leaf fragments with whole galls were stained with Trypane blue, and phloroglucinol with hydrochloric acid (lignin detection). After staining, the slides were mounted on microscope slides in 50% glycerol. The anatomical observations were performed using an Olympus microscope (BX53 and BX61 with fluorescent unit) and Olympus SC30. Micrographs were taken using CellSense Standard program.

## Biochemical analysis

### Hydrogen peroxide (H_2_O_2_) determination

The level of H_2_O_2_ was measured till 2 hour after leaflet collection from single tree. The content of H_2_O_2_ was determined according to the ferrithiocyanate method of [42]. The H_2_O_2_ content of was expressed as nM g^− 1^ FW.

### Antioxidant enzymes assay

All extraction procedures were conducted at 4 °C. Samples were ground in liquid nitrogen and homogenized in extracting buffer.

*The glutathione peroxidase* (GPX, EC. 1.11.1.9.) was conducted as described by [43]. GPX activity was expressed as nM of NADPH min^− 1^ mg^− 1^ protein.

*Catalase activity* (EC. 1.11.1.6) measurements were done according to [44]. The results were shown as nM min^− 1^ mg^− 1^ protein.

*Protein content* was determined according [45].

### Protein and non-protein thiol levels

For determination of thiol protein level, we use [46] method. Protein bound thiols were calculated by subtracting the non-protein thiols from total thiols. A standard curve was prepared using glutathione (GSH) (Sigma-Aldrich, Steinheim, Germany), and thiol contents were expressed as glutathione equivalents.

### Lipid peroxidation

Products of lipid peroxidation as thiobarbituric acid (TBA) reactive substances (TBARS) were measured with method describes by [47]. The level of TBARS was shown as the difference of the absorbance’s A_532_–A_600_ mM g^− 1^ FW.

### Analysis of phenolic compound

#### Phenols free and bond

The samples were extracted with 90% ethanol and heated in a boiling water bath according to method described by [48] (modified). After extraction, the ethanol extracts were evaporated in vacuum and a certain volume of water (approx. 1 mL was added).

#### Determination of free phenols

1 mL of water extract was made up to 7 mL with water. Next 0.5 mL of Folin reagent was added and mixed. After 3 min, 1 mL saturated Na_2_CO_3_ was added to the sample and mixed. The absorbance was measured spectrophotometrically after an hour at 725 nm.

#### Determination of phenols bound

To 2 mL of aqueous extract 2 mL of 2 M HCl were added. Then the samples were heated on a water bath at 90 °C for 20 min. Next the probes were neutralized with 2 M NaOH and the absorbance were measured using spectrophotometer at 725 nm.

### Estimation of tannin content

Tannin content was determined according to [49]. Total tannins content shown as mg GAE 100 g^− 1^ DW.

### Determination of plant pigments

Plant pigments (chlorophylls and carotenoids) were extracted from leaves using methanol (99.9%) [50]. Tissues were homogenized using the TissueLyser LT (QIAGEN, Germany) and clarified using 0.45 μm, PTFE, HPLC syringe cartridge filters (Scientific Resources Inc., Eatontown, NJ, USA). Agilent 1260 Infinity Series, USA HPLC system with autoinjector (500 μL sample loop), refrigerated autosampler compartment, thermostatted column compartment, quaternary pump with in-line vacuum degasser, and photo-diode array detector set to monitor visible spectra from 350 to 700 nm was used for pigment studies. Eclipse XDB C8 column (150 × 4.6), kept at 25 °C with a column oven, and was used for pigment separation and analysis. Eluent A was a mixture of methanol/acetonitrile/0.25 M aqueous pyridine (pH 5.0) solution (50/25/25, v/v/v), while eluent B was methanol/acetonitrile/acetone (20/60/20, v/v/v). The gradient (1 mL min^− 1^) was linear from the specified initial percent solvent A to 100% solvent A from the 1st to 40th minute. The analytical data were integrated using ChemStation software for LC systems. The contents of individual photosynthetic pigments were calculated as μg g^− 1^ FW.

### Determination of monosaccharides

The monosaccharide content presents in the fresh weight of plant leaves was estimated after extraction with ethanol during 24 hours according to [51]. Amount of monosaccharides was shown as mg g^− 1^ FW.

### Determination of fatty acids

For fatty acids determination, plant leaves (0.1 g) were extracted with 0.5 mL of hexane in the presence of 1% methanol-potassium hydroxide mixture as a catalyst in transesterification reaction to obtain FAMEs [52]. The extraction was assisted by an ultrasonic cleaning bath (Sonorex Digital 10P, Germany) at 60 °C for 90 min. Then three portions of 1 mL of hexane were added and the solvent was evaporated under a gentle stream of nitrogen. The residue was redissolved in 1 mL of hexane and 1 μL of this solution was injected for GC/MS analysis. The FAMEs were analysed by a gas chromatograph (7890B GC System) with a mass selective detector MSD5977A (Agilent Technologies, USA). The samples (1 μL) were injected via a Agilent 7683 Injector and Sample tray Series with split ratio 30:1. The injector and transfer line temperatures were kept at 260 °C. A Select HP-88 capillary column (100 m × 0.25 mm, 0.20 μm, 5 in. cage) (Agilent Technologies, USA) and helium as carrier gas (1 ml min^− 1^) were used. The GC temperature program started at 140 °C (hold time 5 min) and was increased to 240 °C at a ramp rate of 4 °C min^− 1^. The electron energy was 70 eV and the temperature of the ion source was set to 250 °C. Retention times (RT) and fragment ions including *m/z* 55.1, 67.1, 79.1, 74.1, 81.1, 87.1, and 99.1 for FAMEs were recorded throughout the run.

### Statistical analysis

The data were analysed with Statistica (Statsoft Poland). For the statistical analysis of the biochemical results, we used ANOVA and Duncan post-hoc test, *p* < 0.05. The mean values ± S.E were shown in each figures.

## Results

### Anatomy of *R. pseudoacacia* leaflet

**Fig. 2:**
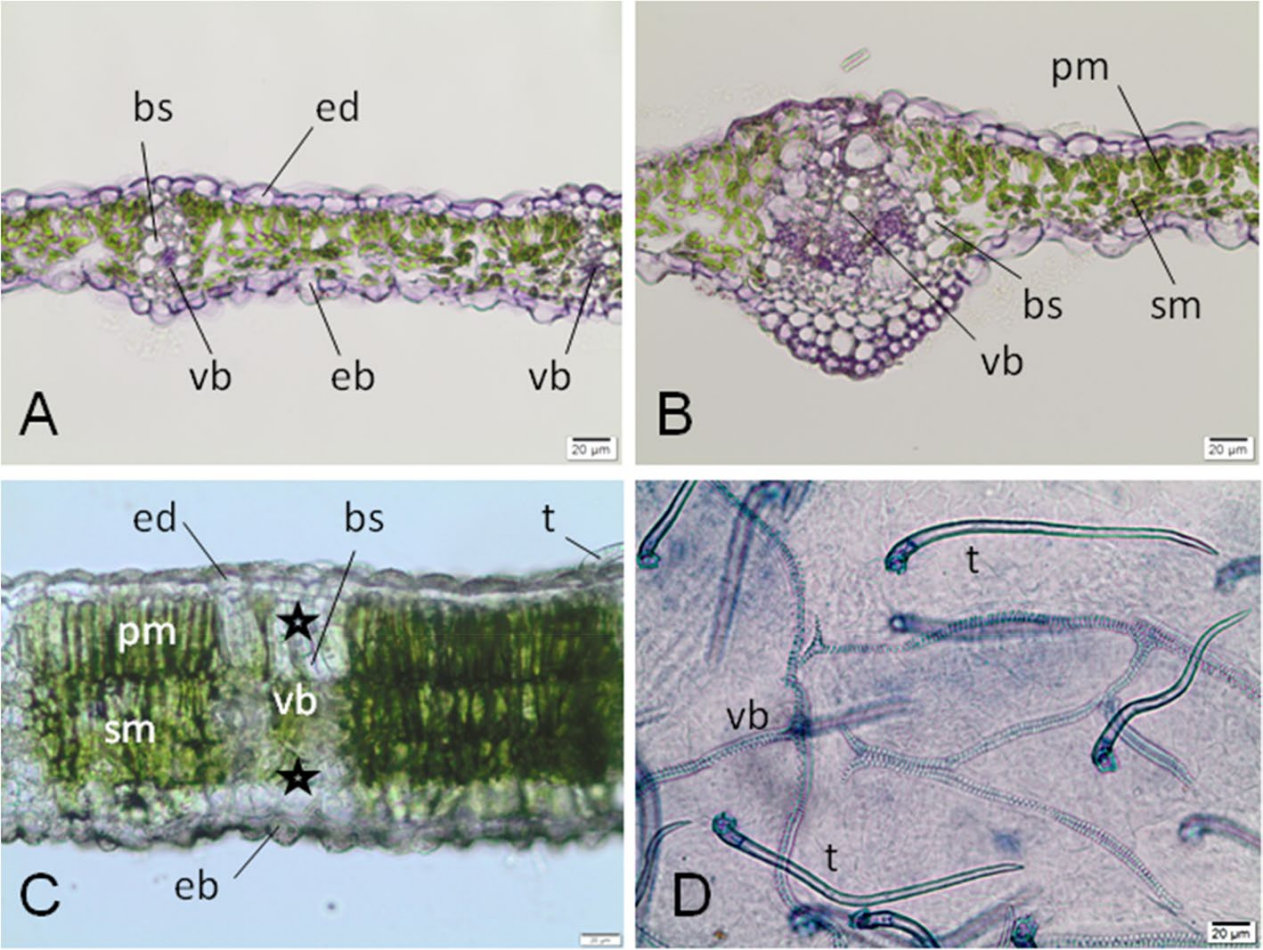
The leaf blade of *R. pseudoacacia* besides a gall (control). **a** Cross section through a young fresh leaf blade in their side part, with small vascular bundles; cryostate section, stained with Ehrlich hematoksylin. **b** Cross section through the same, young fresh leaf with palisade and sponge mesophyll and a bigger (no central) vascular band; cryostate section; stained with Ehrlich hematoksylin. **c** Transverse, free hand section of older, mature fresh leaf of *O. robiniae*, no stained, with a mesophyll (palisade and spongy), vascular bundles enclosed by bundle sheaths consisting of almost colourless parenchyma (stars). Bundle sheaths extensions extend from the veins to the upper and lower epidermis. The layer of parenchyma with small amount of chloroplasts extends under lower epidermis. **d** Part of the whole cleared leaf from the side of abaxial epidermis; many mechanical trichomes and reticulate venation are visible; slide stained with Trypane blue. Abbreviations: eb - abaxial (lower) epidermis, ed. - adaxial (upper) epidermis, mp - palisade mesophyll, ms - spongy mesophyll, vb - vascular bundle, t - trichome, bs - bundle sheath, * colourless parenchyma. Bar = 20 μM

A *R. pseudoacacia* leaf is bifacial, dorsiventral and hypostomatous. The leaf blade of the young leaves is thin and thickens during maturation (Fig. 2a-b). At the lower epidermis stomata and trichomes (Fig. 2d) were notice, in opposite to upper epidermis where we faund flatter cells and few trichomes. The mesophyll of the young leaf consists of one layer of palisade mesophyll and a 2–3 cells layer of spongy meshophyll (Figs. 2a-b). Only small and medium-sized vascular bundles (Fig. 2a-c) appear in the marginal part of the leaf. This structure is surrounded by bundle sheaths composed of almost colorless parenchyma of one to several cells (Fig. 2c). A parenchyma layer with a small amount of chloroplasts is located under the lower epidermis (Fig. 2c). The vascular bundles are accompanied by fine calcium oxalate crystals.

**Fig. 3:**
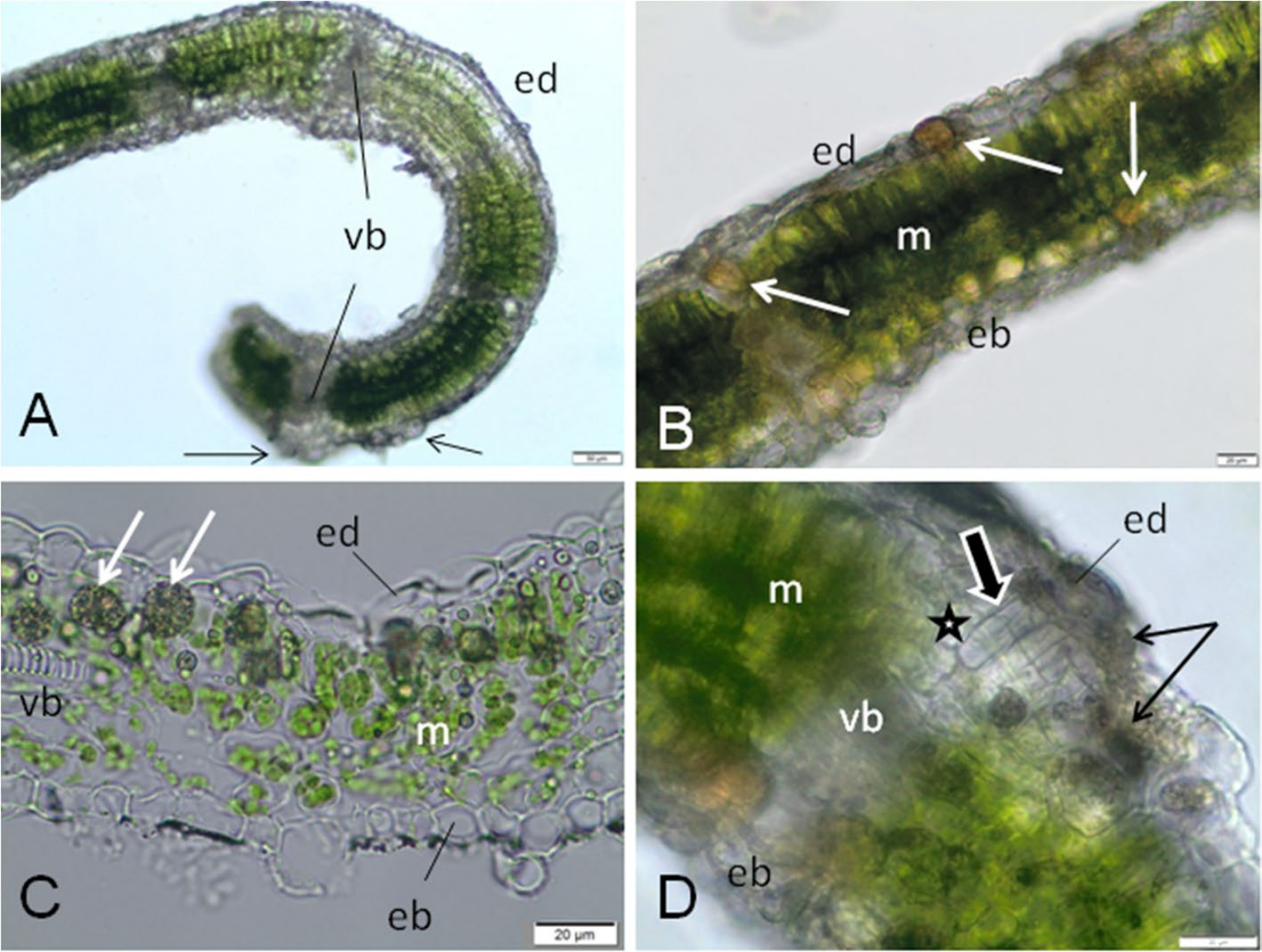
Early stage of development of *O. robiniae* gall on the *R. pseudoacacia* leaves. **a**, Bar = 50 μM) Transverse section through a bent edge of fresh leaf; the thickness of the leaf blade in the curved part is the same as in the rest one; some cells in adaxial epidermis are enlarged (arrows); free hand section, no stained. **b**, **c**, **d**; Bar = 20 μM) (**b**) Transverse section through a initiating gall; inside of some leaf cells any pigmented (orange-brown) material is visible (arrows); fresh section, no stained. **c** Hydrophobic, heterogeneous material in form of balls (arrows) in leaf cells, mainly in mesophyll; cryostate section from fresh leaf, no stained. **d** Part of the wall of a initiating gall; fibrous material similar to fungi in epidermis and parenchyma of leaf cells (arrows). The cells of parenchyma in the bundle sheath extension (star) are divided longwise and transversely (bold arrow); free-hand section, no stained. Abbreviations: eb - abaxial epidermis, ed. - adaxial epidermis, m -mesophyll, vb - vascular bundle, * bundle sheath

During the gall initiation phase, the edge of the leaf blade prior to gall formation is green with small to medium veins (Fig. 1e). After gall induction, the entire edge of the leaf visibly curls. At this stage, the thicknesses of the whole leaf (in its folded and erect portions) are similar (Fig. 3a). The upper epidermis becomes the outer surface of the gall; thus the part of the cells of this tissue is enlarged (Fig. 3a). Orange-brown pigments are visible in some cells of the upper epidermis and mesophyll (Fig. 3b). Large, spherical clusters of heterogeneous and hydrophobic material (probably with lipid), mainly in the palisade mesophyll, are observed in other galls at an early stage of their development (Fig. 3c). Sometimes, granular-fibrous materials are present in epidermis and mesophyll cells resemble fungi in appearance (Fig. 3d), but do not stain in reaction with the Trypane blue used for fungal detection. The parenchyma cells of the bundle sheath extension, below the upper epidermis, are divided (Fig. 3d).

**Fig. 4:**
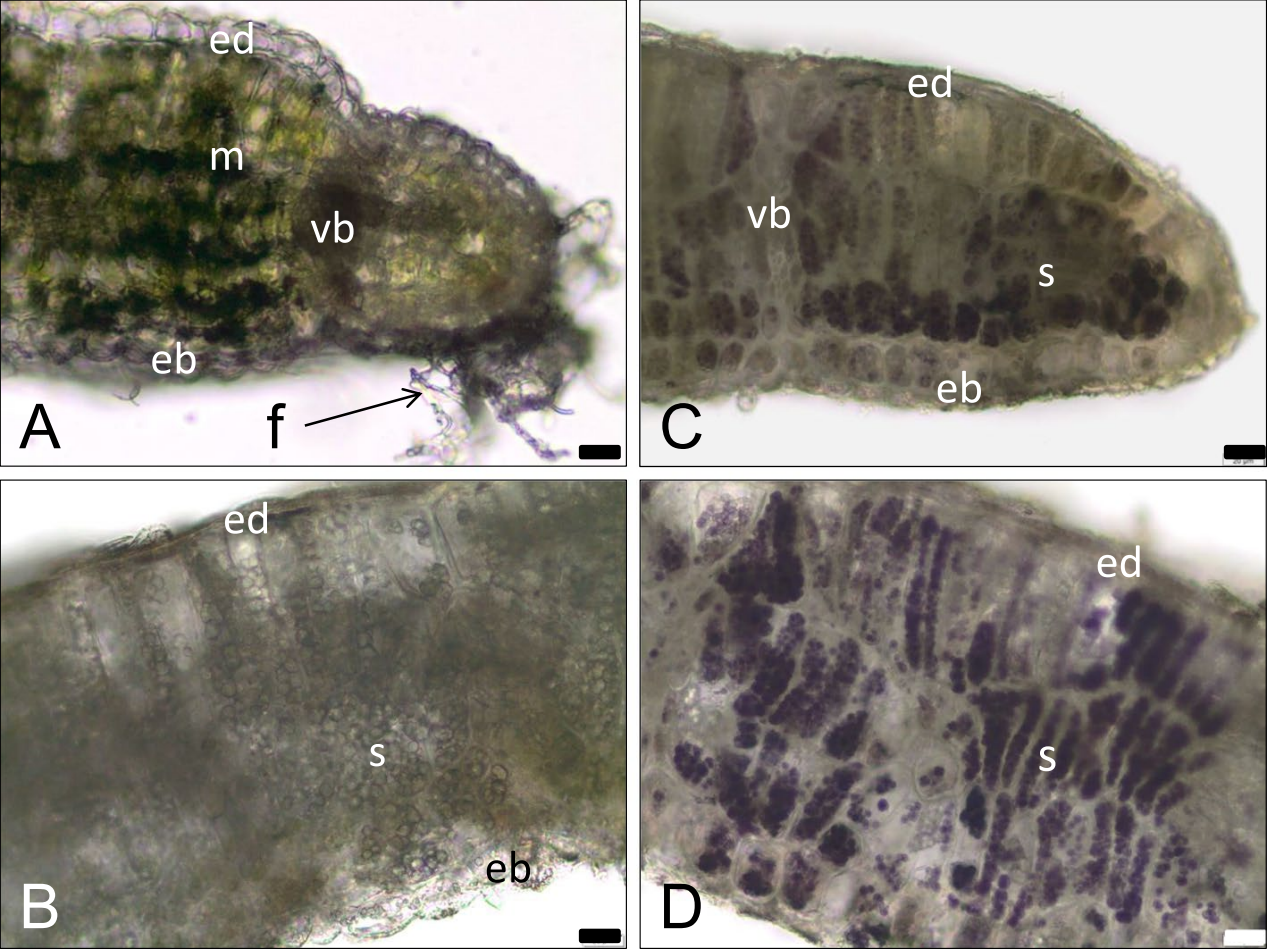
Mature, yellowish galls of *O. robiniae* with starch**,** sections through a galls in fresh leaves. **a** Part of the leaf transforming into a gall (maturation phase). The cells of adaxial epidermis are colourless and clearly convex. Mesophyll cells are enlarged, with chloroplasts containing starch. The hyphae (arrow) on the edge of the leaf are visible. **b** Thicked wall in the central part of the mature gall. Whole mesophyll has been transformed into the colourless storage parenchyma (nutritive tissue) with amyloplasts containing big starch grains; free hand section, no stained. **c**, **d** Sections of the gall wall with starch after reaction with potassium-iodide (Lugol liquid), starch is stained on violet. **c** Section from the margin part of a gall wall. The cells of external epidermis (ed) are flattened and stretched. Not all cells of nutritive tissue are evenly filled with starch. **d** Section from the central part of the gall wall. The cells of nutritive tissue are tightly filled with starch, what well reveals the cell shapes. Abbreviation: eb - abaxial epidermis, ed. - adaxial epidermis, lc - larval chamber, m - mesophyll, s - starch, f - fungi (hyphae), vb - vascular bundle. Bar = 20 μM

The wall of the mature gall is thicker than the leaf from which it was formed and yellow-green due to the reduction of chlorophyll (Fig. 1f). The gall-forming leaf fragment changes its function from assimilation to protective. The mesophyll is completely replaced by parenchyma (nutrient tissue of large cells filled with starch plastids) (Fig. 4b-d), with a more compact cell layout and with thicker cell walls. The cells of the outer gall epidermis are flattened and stretched (Fig. 4b-d). The storage tissue is built from only a few cells, many of which are narrow and elongated (Fig. 4d). On preparations with starch stained with Lugol’s solution, the shapes of the cells of the nutrient tissue and their compact arrangement are clearly visible (Fig. 4c-d). Not all of the starch is stained with the same intensity and not all cells are evenly filled with starch (Fig. 4c-d).

**Fig. 5:**
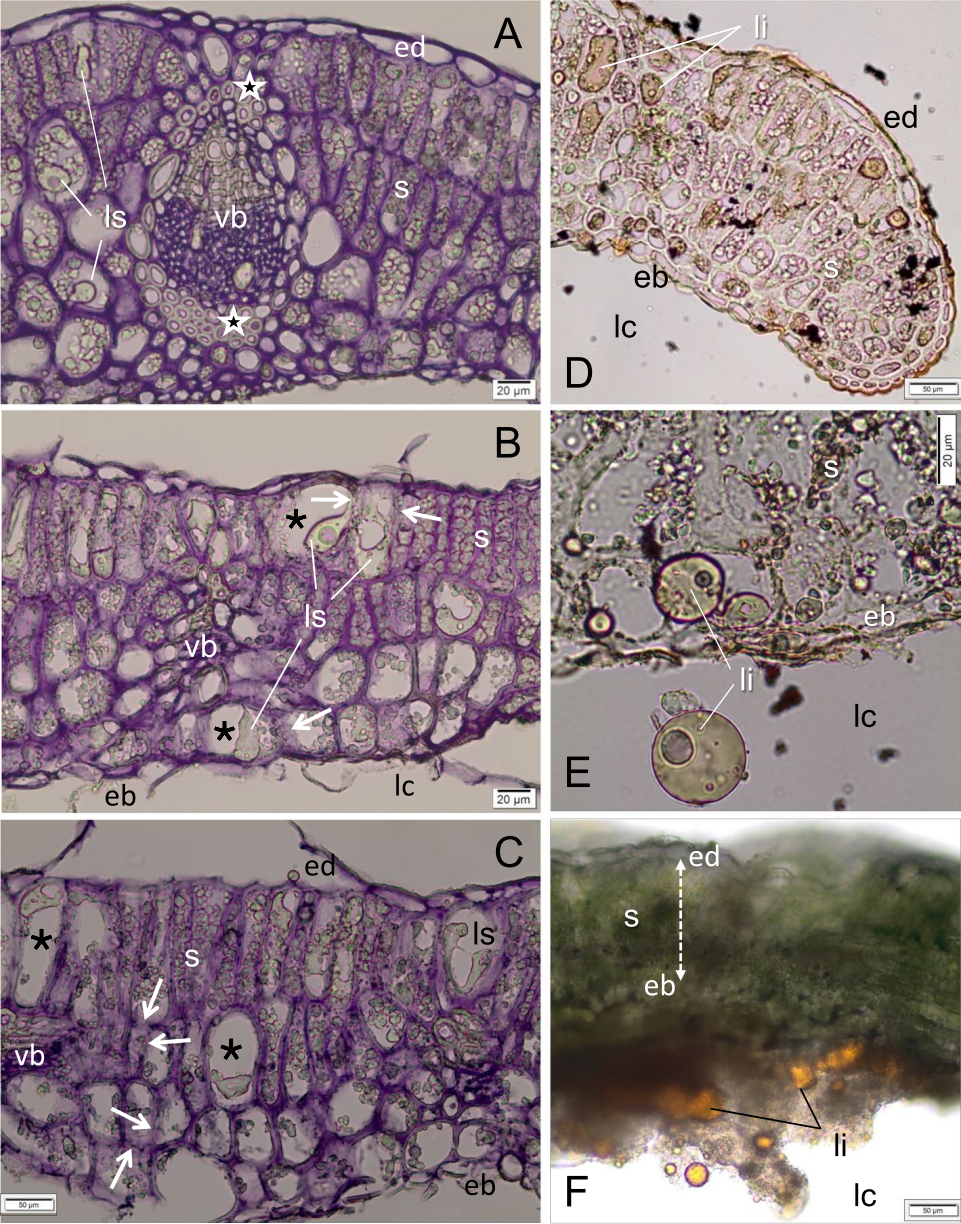
Mature, yellowish galls of *O. robiniae* with starch and lipids. **a**-**c** progressive changes in the nutritive tissue of the gall; transverse, cryostate sections stained with Ehrlich hematoksylin. Cell walls of nutritive tissue, stained on violet, are clearly bold. The cells contain both starch grains (s) and additionally liquid material (ls). **a** Note the sclereids belonging to the bundle sheats (white stars) around the bigger vascular bundle. **b** In enlarged cells of nutritive tissue (black stars), where the starch disappeares, a liquid material (ls) next to the partly dissolved the cell wall with adjacent cells (white arrows). Epidermis (eb) from the larval chamber side is damaged. **c** Groups of the adjacent cells with partly dissolved the cell wall (black arrow). The gall wall from the side of larval chamber (epidermis and parenchyma) is strong damaged. **d**, **e**) Cryostate sections (from the same galls as show in Fig. 5a-b) in reaction with Sudan III: cuticle on epidermis and lipids (li) in nutritive tissue are stained orange. **e** Lipid droplets (li) from the gall nutritive tissue with admixture of another substances are visible. **f** Transverse section, not dyed, through the mature gall with starch in nutritive tissue (between two epidermis) and lipids (li) in the larval chamber (free hand section, a little thick). Abbreviation: eb - abaxial epidermis, ed. - adaxial epidermis, lc - larval chamber, li - lipids, ls -liquid substances, s - starch, vb - vascular bundle. (**a**-**f**, Bar = 20 μM), (**g**, Bar = 50 μM)

Some mature galls, morphologically identical to those with starch, present an altered image of the storage tissue (Figs. 5a-c). The cell walls are thickened, the starch grains swollen, and some cells additionally contain liquid material (Fig. 5a). In slightly older galls (Fig. 5b-c) the amount of starch in the nutrient tissue decreases (the cells contain significantly less starch grain and some are optically empty), and in many cells there is some liquid material next to the locally dissolved cell wall. Groups of storage parenchyma cells with partially dissolved cell walls are visible in various parts of the gall wall: at the vascular bundles, under the upper epidermis, or from the side of the larval chamber, directly under the damaged lower epidermis (Fig. 5b-c). Sections with the same galls as shown in Fig. 5a-b, but treated with Sudan III reagent, reveal the presence of lipids in the cells of the nutrient tissue (Fig. 5d). In the magnified image, admixtures of other materials are visible in the lipid droplets from the storage tissue (Fig. 5e). High amounts of lipids and admixtures of other materials in the larval chamber are observed on the freehand sections of fresh galls (Fig. 5f).

**Fig. 6:**
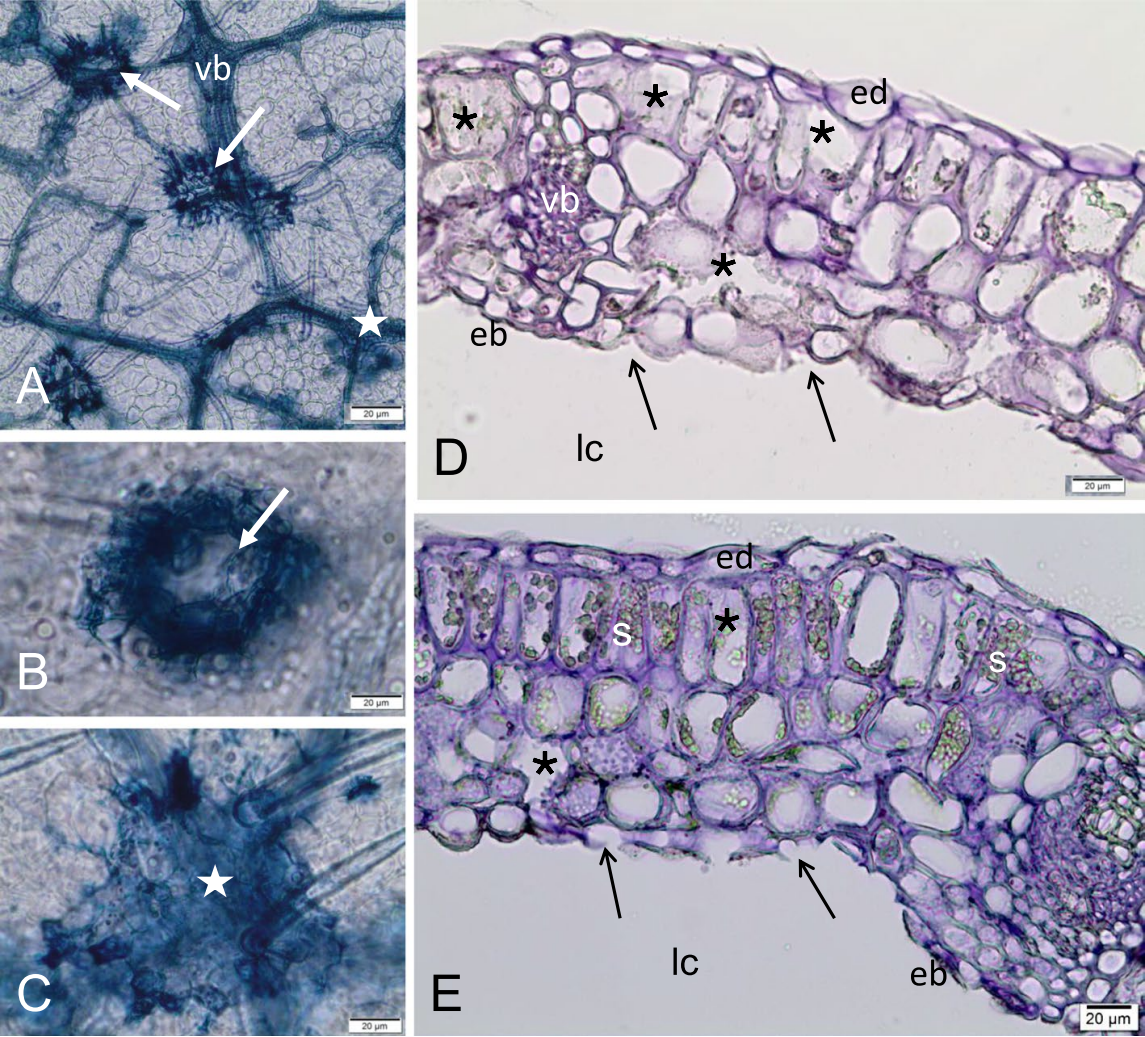
Signs of *O. robiniae* larvae feeding in a galls. **a**-**c** Part of the cleared gall wall, stained with Trypane blue; view from the larval chamber side. **a** Damages of abaxial epidermis in form of craters (arrows) and surface abrasions (star) next to the vascular bundles. **b** Enlarged place of destroyed epidermal cells in form of crater (arrow). **c** Enlarged place of surface abrasion of epidermis. **d**, **e** Transverse, cryostate sections through the mature gall wall damaged by feeding of *O. robiniae* larvae; fresh material, stained with Ehrlich hematoxylin. Abaxial epidermis from the larval chamber side is broken and in some places absent (arrows). **d** The cells of nutritive tissue are optically almost empty. The groups of neighbouring cells with partly dissolved cell walls are visible (stars). **e** Similar image as in Fig. 6d, but here part of the gall wall with less destroyed structure. In the nutritive tissue the groups of cells with dissolved cell walls (stars), and also the intact cells with starch (s) in plastids are visible. Bar = 20 μM

**Fig. 7:**
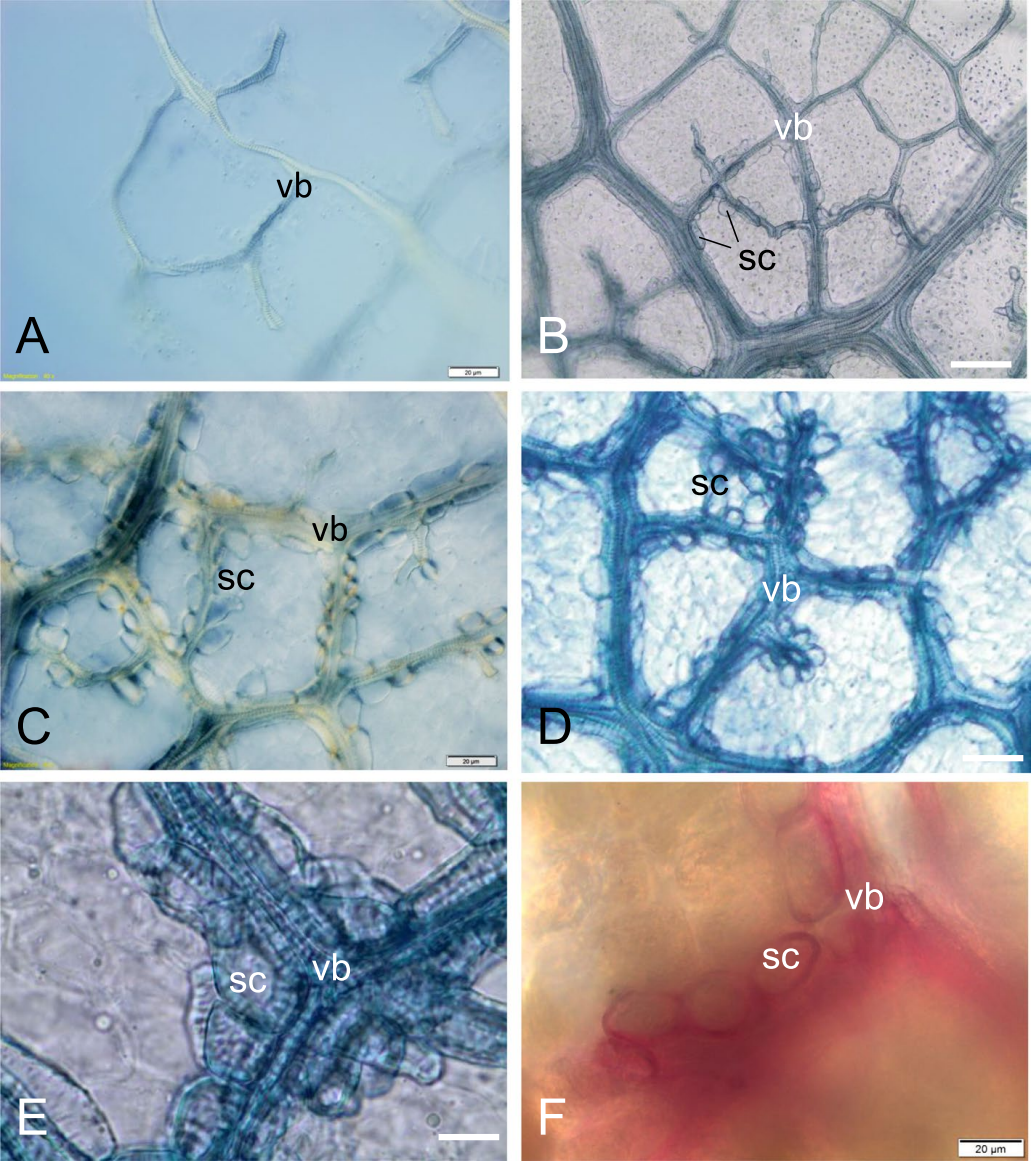
Sclerenchyma in the wall of mature galls induced by *O. robiniae*. **a** Control. Network of the vascular bundles in robinia leaf, outside the gall; part of the cleared leaf; view in Nomarski contrast. **b** Part of the cleared gall, stained with Trypane blue. The boundary between the normal leaf (upper part) and the gall (lower part) is visible. In the area of gall the vascular bundles are surrounded by sclereids (sc). **c** Cleared part of the mature gall; vascular bundles are surrounded by one layer of sclereids; view in Nomarski contrast. **d** Middle part of the mature gall wall; sclereids are present around all small vascular bundles; specimen stained with Trypane blue. **e** Magnified image of the vascular bundle enclosed immediately by sclereids. It is clear, that sclereids (brachysclereids) are lignified cells of parenchyma bundle sheath. Note in sclereids the thick call wall containing numerous simple pits; specimen stained with Trypane blue. **f** Specimen of the mature gall wall stained with phloroglucinol and HCl; lignin in sclereids and tracheary elements is stained red. Abbreviations: sc - sclereid, vb - vascular bundle. Bar = 20 μM

In this gall, around the conductive bundles, sclereids (lignified cells of the parenchyma) of the bundle sheath are visible (Fig. 5a); in the leaf outside the gall the bundle sheath is non-lignified (Fig. 2b-c). The epidermal changes are visible mainly around the vascular bundles (Fig. 6a). The lesions form craters (circular areas of damaged epidermis), sometimes extending into the storage tissue (Fig. 6b). Injured tissues are stained intensely when treated with Trypane blue (Fig. 6a-c), as are the vascular bundles. The changes inside the galls concern the cell walls and storage material in the nutrient tissue. In older galls, most of the cells of the feeding tissue are nearly optically empty (without storage materials), and partially dissolved cell walls (Fig. 6d-e) are observed. The gall wall is stiffened by a sclerenchyma, which prevents them from collapsing and protects the larvae feeding inside. It is present in both the control leaves (Fig. 7a) and in young developing galls, but it only appears in those that have reached their final size and is present only in the galls, as is clearly visible in the leaf/gall transition zone (Fig. 7b). Sclerenchyma is strongly reduced here, because it does not form a uniform layer in the gall wall, but an openwork structure limited to stone cells (brachysclereids) surrounding the vascular bundles. Sclereids are formed by lignification of the parenchyma cells of bundle sheaths (Fig. 7b-f), and surround small veins with a single layer (Fig. 7c-e), or form larger groups of cells at larger vascular bundles (Fig. 5a). Straight pits are visible in the thick walls of the sclereids (Fig. 7e), as confirmed by reaction with phloroglucinol and HCl (Fig. 7f).

**Fig. 8:**
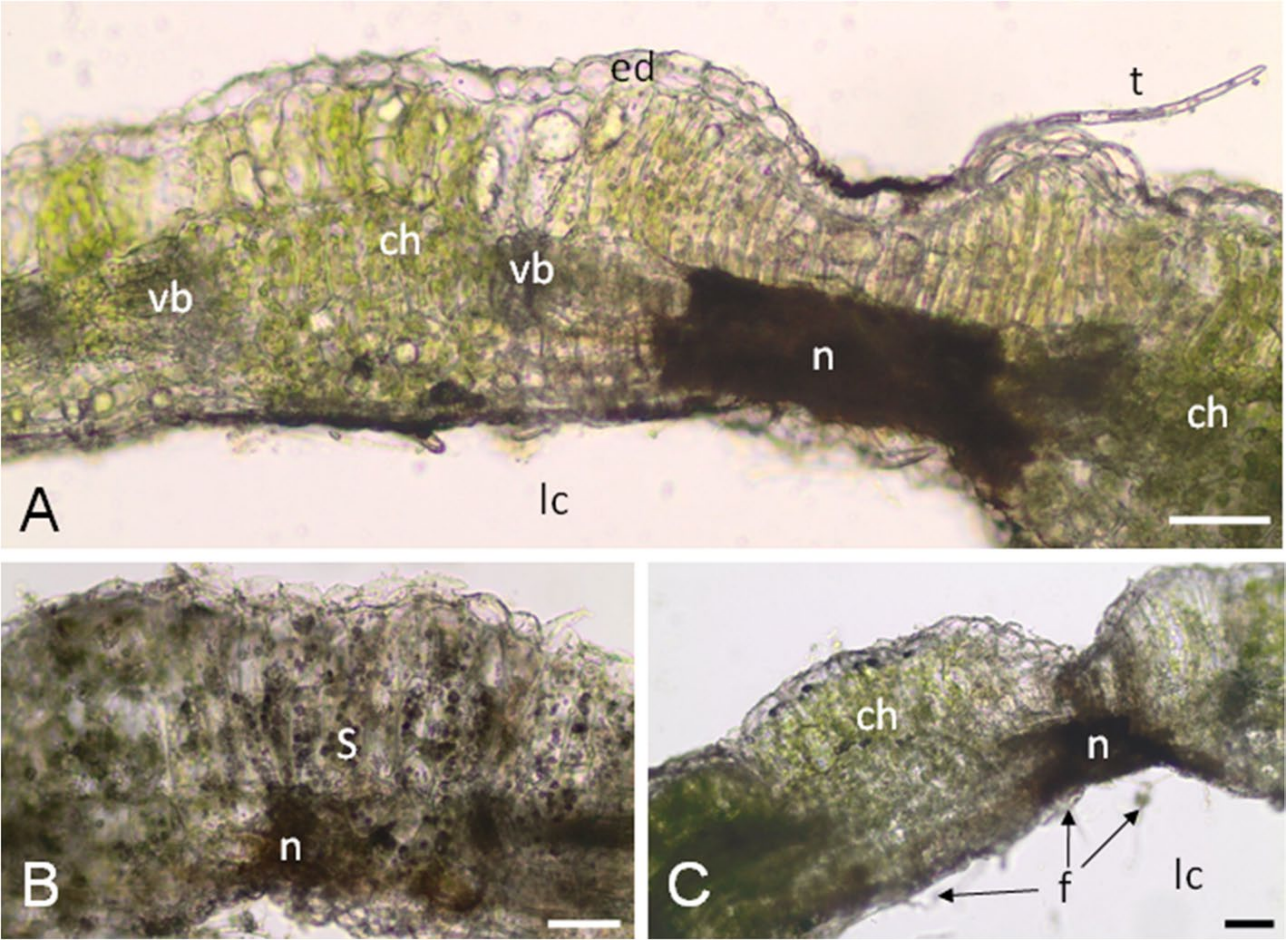
Aging galls of *O. robiniae* on the *R. pseudoacacia* leaves. **a** Transverse, free hand section through the wall of aging gall; fresh material, no stained. Many chloroplasts are visible. Necrotic, dark stained and compact area (n) is located from the side of larval chamber (lc); Bar = 50 μm. **b** Chloroplasts are rich in starch (s); starch grains on this section are stained violet with Lugol liguid; Bar = 50 μM. **c** Inside the larval chamber, on the internal surface of the gall wall many hyphae are visible (f); Bar = 50 μM. Abbreviations: ch - chloroplast, f - fungi (hyphae), lc - larval chamber, n - necrotic area, s - starch, vb -vascular bundle

Aging galls, abandoned by insects, are easily recognizable by dark brown necrotic spots (Fig. 1d), which do not show any structure on microscopic sections (they are groups of cells filled with dark material), which are located on the side of the larval chamber (Fig. 8a-c). The galls at this stage are also greener than in the mature stage (Fig. 8a-c) and contain chloroplasts with a large amount of starch (Fig. 8b). In the larval chamber, on the inner surface of the gall wall, fungal hyphae are present in mature (Fig. 4a) and aging (Fig. 8c) galls.

### Metabolic processes

**Fig. 9:**
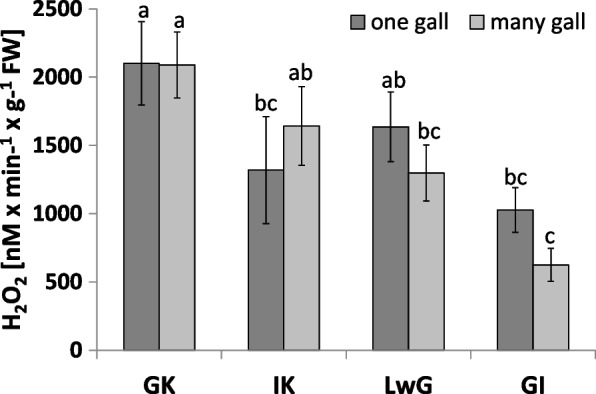
Level of hydrogen peroxide (H_2_O_2_) in leaflets of *R. pseudoacacia* infected by *O. robiniae*. Results of analysis made on leaflets infected by one or many galls. Global control - GC, internal control - IC, leaflets with gall - LwG, leaflet after gall isolation - LI, isolated gall - GI. Statistically significant differences are indicated with different letters according to Duncan Test when *p* ≤ 0.05. The data are the means ± SD of four biological replicates

Since no statistical accordance was noticed between one or many galls per leaflet in relation to the level of hydrogen oxide (Fig. 9), further results were showed without dividing leaflets into this kind of class.

### Oxidative parameters and phenol content

The level of H_2_O_2_ was modified in response to insect attack. The highest content was shown in leaves that had no sign of gall formation, treated as global control. A similar level of H_2_O_2_ was observed in internal control and leaflets with gall as opposed to variants of isolated galls where H_2_O_2_ level was the lowest (Fig. 9).

**Fig. 10:**
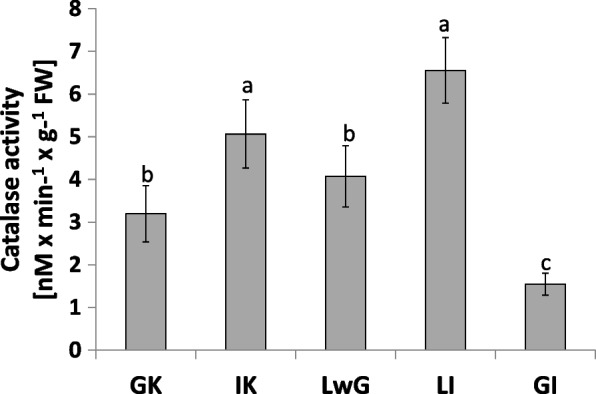
Catalase activity in leaflets of *R. pseudoacacia* with gall formed by *O. robiniae*. Global control - GC internal control - IC, leaflets with gall - LwG, leaflet after gall isolation - LI, isolated gall - GI. Statistically significant differences are indicated with different letters according to Duncan Test when p ≤ 0.05. The data are the means ± SD of six biological replicates

The strongest catalase activity was observed in leaflets after gall isolation. In isolated gall structure, it was the least active (Fig. 10). In leaflets from internal control, catalase activity was stronger than in global control and leaflets with gall.

**Fig. 11:**
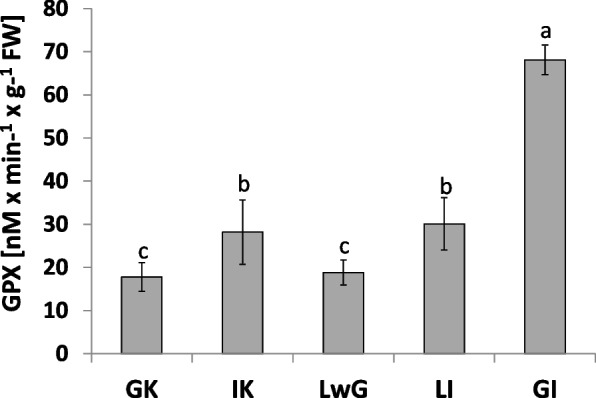
The activity of glutathione peroxidase (GPX) in leaflets of *R. pseudoacacia.* Changes in activity of gluthathione peroxidase (GPX). Global control - GC, internal control - IC, leaflets with gall - LwG, leaflet after gall isolation - LI, isolated gall - GI. Statistically significant differences are indicated with different letters according to Duncan Test when p ≤ 0.05. The data are the means ± SD of four biological replicates

Glutathione peroxidase (GPX) (Fig. 11) was comparably active in internal control and leaflets after gall isolation but was more active in comparison with in global control and leaflets with gall structures. Gall structures were characterized by the strongest GPX activity.

**Fig. 12:**
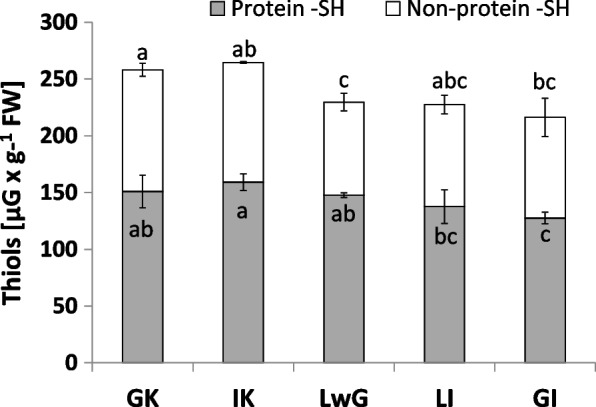
Thiol content in leaflets of *R. pseudoacacia* with gall formed by *O. robiniae*. Global control - GC, internal control - IC, leaflets with gall - LwG, leaflet after gall isolation - LI, isolated gall - GI. Open bars means non-thiol proteins with Duncan Test letters, close bars means protein thiol with Duncan Test letters. Statistically significant differences are indicated with different letters according to Duncan Test when p ≤ 0.05. The data are the means ± SD of four biological replicates

Differences in levels of thiol and non-thiol proteins were revealed. In both the control variants, the level of the non- protein thiols was the highest during the experiment. On the other hand the lowest value was notice in leaflets with gall (Fig. 12). The thiol protein level was highest in internal control, whereas its content in global control and leaflets with gall was comparable (Fig. 12).

**Fig. 13:**
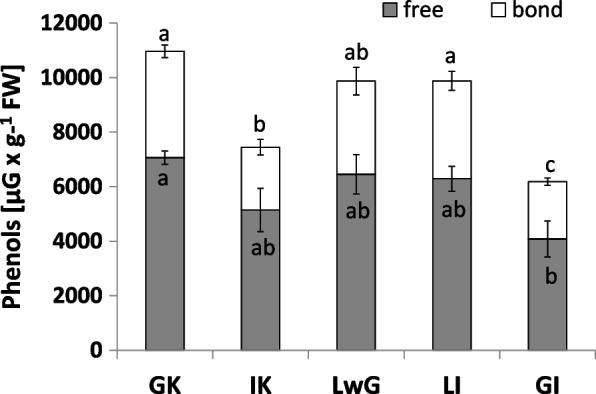
Phenols content in leaflets of *R. pseudoacacia* with gall formed by *O. robiniae*. Phenols content shown as bound an free in different variants. Global control - GC, internal control - IC, leaflets with gall - LwG, leaflet after gall isolation - LI, isolated gall - GI. Statistically significant differences are indicated with different letters according to Duncan Test when p ≤ 0.05. The data are the means ± SD of four biological replicates

Changes in patterns of phenol level were demonstrated in the tested samples. The lowest content of free phenols were noted in GI, whereas the levels of bound phenols in GC and in LI were the highest. Their amount in IC was lowered, and the smallest ration was notice in GI structure (Fig. 13).

**Fig. 14:**
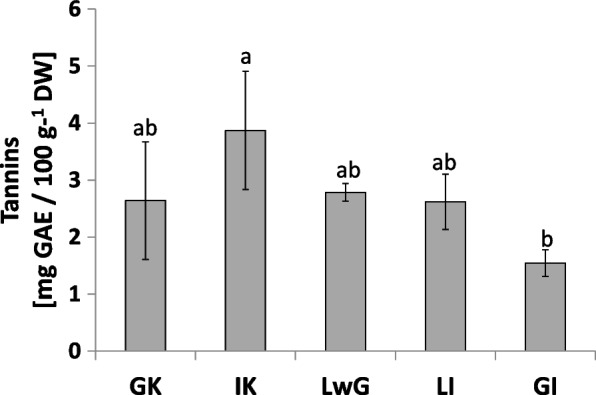
Tannins content in leaflets of *R. pseudoacacia* with gall formed by *O. robiniae*. Global control - GC, internal control - IC, leaflets with gall - LwG, leaflet after gall isolation - LI, isolated gall - GI. Statistically significant differences are indicated with different letters according to Duncan Test when p ≤ 0.05. The data are the means ± SD of four biological replicates

The level of tannins was higher in internal control than in other treatments. A lower value was demonstrated in gall structure, whereas it was at a similar level (Fig. 14) in all analyzed variants (GC, LWG, LI).

### Pigments and monosaccharides content

#### Monosaccharide content

The highest contents of monosaccharides were observed in the controls, especially in the internal control (Table 1). During gall formation a gradual decrease in the monosaccharide level was noted, reaching its lowest content in leaves with aging gall.

**Table 1:** Content of monosaccharides and plant pigments during different stages of *O. robiniae* gall formation

Content of monosaccharides [mg g^**− 1**^ FW] and plant pigments [μg g^**− 1**^ FW]	Controls and plants with different stage of gall formation				
	Global control	Internal control	Young gall	Mature gall	Aging gall
**Monosaccharides**	8,7 ± 1,02^b^	10,68 ± 1,3^a^	6,47 ± 1,14^c^	5,13 ± 0,68^d^	4,15 ± 0,9^e^
***Chlorophyllide a***	16.25 ± 2.21^a^	15.36 ± 1.84^b^	8.73 ± 3.91^c^	8.27 ± 2.06^c^	4.47 ± 1.82^d^
***Chlorophyllide b***	10.18 ± 6.74^a^	4.44 ± 0.29^b^	3.41 ± 1.72^c^	4.94 ± 0.2^b^	1.42 ± 0.36^d^
**Chlorophyll** ***a***	519.39 ± 66.95^a^	497.53 ± 22.17^b^	482.36 ± 31.04^b^	143.56 ± 21.53^d^	183.73 ± 11.35^c^
**Chlorophyll** ***b***	164.09 ± 17.56^a^	152.32 ± 15.49^a^	115.39 ± 10.05^b^	54.31 ± 12.35^c^	90.76 ± 8.46^b^
***α*-Carotene**	11.76 ± 4.21^b^	13.24 ± 0.43^b^	6.55 ± 0.41^c^	5.22 ± 0.76^d^	17.32 ± 2.88^a^
***β*-Carotene**	30.09 ± 5.92^c^	46.40 ± 5.28^b^	23.66 ± 4.11^d^	26.72 ± 2.54^d^	55.95 ± 7.54^a^
**Astaxanthin**	4.56 ± 0.99^c^	4.11 ± 1.08^c^	6.54 ± 1.12^b^	5.99 ± 0.67^b^	9.62 ± 1.38^a^
**Neoxanthin**	3.85 ± 0.28^c^	2.34 ± 0.59^d^	3.77 ± 1.03^c^	4.58 ± 0.17^b^	5.22 ± 0.44^a^
**Cryptoxanthin**	6.96 ± 0.77^c^	7.57 ± 0.55^c^	8.98 ± 0.76^b^	12.15 ± 0.78^a^	13.66 ± 1.63^a^
**Lutein**	2.74 ± 0.33^c^	2.68 ± 0.15^c^	2.36 ± 0.44^d^	3.93 ± 0.22^b^	4.65 ± 0.43^a^
**Violaxanthin**	20.72 ± 1.35^c^	22.75 ± 3.27^c^	25.35 ± 4.48^b^	23.91 ± 3.44^b^	35.77 ± 2.11^a^
**Zeaxanthin**	31.48 ± 2.56^d^	35.37 ± 3.32^c^	36.77 ± 3.33^c^	46.17 ± 2.11^b^	50.22 ± 1.47^a^

Data are the means of three independent experiments ± SE. Treatment with at least one letter the same are not significantly different according to Duncan post-hoc test, p < 0.05

#### The content of plant pigments

During gall formation, some significant decreases in the levels of chlorophylls and their precursors were noted. Internal and global controls were characterized by the highest levels of chlorophyll *a* and *b*. Gall formation caused marked reductions in chlorophylls levels. However, their concentrations were very high at the early stage of gall formation. The lowest value was noticed in leaves with mature gall. Similarly, the greatest increase in chlorophyllide contents were observed in global controls. These plant pigments were shown to be four times fewer in leaves with aging gall.

The gradual decrease in the level of carotenes in *R. pseudoacacia* leaves at early stages of gall formation was observed. On the other hand, leaves with aging galls were characterized by the highest content of *α* and *β*-carotene in comparison with both controls.

The contents of xanthophyll pigments including astaxanthin, neoxanthin, cryptoxanthin, lutein, violaxanthin, and zeaxanthin in global and internal control were similar. The amounts of xanthophylls increased gradually during gall formation. The highest value of level of these photosynthetic pigments occurred in leaves with aging gall.

### Fatty acid content

**Fig. 15:**
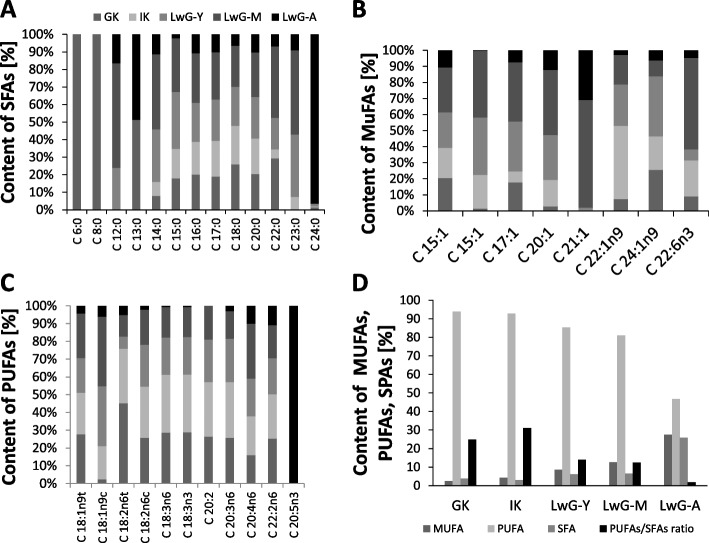
Changes in fatty acid content. The influence of infection of *R. pseudoacacia* leaves with *O. robiniae* on fatty acid content (**a**) saturated, SFA; unsaturated (**b**) monounsaturated MUFA, (**c**) polyunsaturated, PUFA, (**d**) PUFA/SFA ratio. Global control - GC, internal control - IC, leaflets with young gall - LwG-Y, leaflet with mature gall - LwG-M, leaflet with ageing gall - LwG-A

The profiles and levels of saturated (SFA), and unsaturated (MUFA and PUFA) fatty acids are shown in Fig. 15a-d. Leaves with galls were characterized by higher contents of MUFA and SFA. However, a decrease in the level of PUFA was observed in leaves after infection. The highest PUFA/SFA ratio was shown in leaves from internal control, while the lowest level of this proportion was noted for leaves with galls. Most of FAs compounds are present in higher concentration in leaves with galls in comparison with both controls. Among all identified SFAs, two of them (C6:0 and C8:0) were recognized only in global control, while one fatty acid C12:0 was present in leaves during gall formation. An increase in MUFA content was observed in leaves after insect attack. The highest content of this type of fatty acid was recognized in leaves with mature galls. Among them, the levels of C21:1 and C22:6n3 were the most stimulated after insect infection. However, the highest concentration of C24:1n9 was observed at the early stage of gall formation. The content of PUFA in *R. pseudoacacia* leaves generally decreased after *O. robiniae* infection. However, among PUFAs, C20:5n3 is the most characteristic fatty acid in leaves with only aging galls. Additionally, the level of fatty acids, such as C18:1n9c, C20:4n6, and C22:2n6 increased during gall formation.

## Discussion

Gall formation of *O. robiniae* influences the physiology and structure of *R. pseudoacacia* leaves. This is first broad physiological study of plant-insect interactions that produce an open gall structure. *O. robiniae* develops gall by reprogramming the growth plan of a leaflet. While the gall structure forms, the lateral part of the leaf blade is involved (Figs. 1-8). *R. pseudoacacia* reacts by folding the edge of leaflet blade downwards during gall structure formation (Fig. 1a-d). This process may start when eggs are left on the abaxial side of the *R. pseudoacacia* leaf blade margins [38]. In other *Cecidomyiidae,* different scenarios are possible – e.g., as a reaction to wounding by feeding larva [1, 30] or due to spatula usage [53]. However, the precise mechanism of egg deposition and the initial events of gall formation require further research. Each stages of the initial plant reaction is fast enough to form shelter (Fig. 1). The size of the gall structure is not correlated with the number of larvae inside. Several authors [53–55] have discussed the development of a special pouch that shields the growing larvae or developing buds, but this kind of pouch was closed. Nutritive tissue develops due to increasing levels of starch followed by lipids (Fig. 5) forming a food base for the growing larvae. Signs of larvae feeding are visible as abaxial epidermis damage to cells with broken walls These changes are consequences of sourcing nutrients due to craters formed inside larvae chambers near a high order vein (Fig. 6a-c). A similar observation for the midrib gall moth *Sorhagenia nimbosus* (Lepidoptera) on *Copaifera langsdorffii* (Fabaceae) was performed where an open gall structure is formed along the main vein [56]. Open galls induced by a distinct generation of aphid *Smynthrodes betae* (Hemiptera) on *Pistacia atlantica* (Anacardiaceae), vary in shape on the leaflets – e.g. the mid-vein or leaflet margin [57]. According to findings by Harper et al. [58] in cinipids at the early stage of larvae development nutritive tissue with enlarged cells filled with lipids surrounds the larva with polytene nucleus and enlarged nucleoli. In the second pattern of endoreduplicated cell enlargement, they break down to smaller nutritive cells [58].

The obtained results indicate that during gall formation *R. pseudoacacia* leaves contain a higher level of MUFAs and SFA but less PUFAs. The presence of two SFAs (C6:0 and C8:0) in global control (Fig. 15a) can be a source of unpleasant odor which protects plants against insects laying eggs [59–61]. The increase in SFA level may be beneficial for the biosynthesis of lignins, which are polymers made by cross-linking phenolic precursors that form key structural materials in the support tissues of most plants. Lignin also confers plant disease resistance by accumulating at the site of pathogen infiltration, making the plant cell less accessible to cell wall degradation [62]. Our results showed that the level of PUFA decreased after *O. robiniae* attack (Fig. 15c) indicating that these fatty acids can be used in larvae feeding as a source of valuable compounds in their diet. This suggestion is consistent with literature data, demonstrating that leaf lipids are sources of essential fatty acids for larvae of two species of Lepidoptera: *Inachis io* reared on *Urtica dioica*, and *Pieris brassicae* reared on *Brassica oleracea*. In both insects, the mayor sources of linoleic (C18:2) and linolenic (C18:3) acids were leaf phospholipids and glycosyl glycerides, respectively. Linolenic acid was the most abundant fatty acid absorbed from the diet in both species and was the predominant fatty acid in tissue glycolipids and phospholipids [63]. Taken together, our findings suggest that gall insects may be able to nutritionally enhance their food source inducing concomitant increases in fatty acids levels, especially MUFA and SFA and associated defense responses. Zhu at al [64]. showed that free fatty acids C18:1 and C18:3 are released from membrane lipids, and are then quickly converted into defense signaling compounds enhancing plant resistance against the hessian fly. C18:3 is a precursor of plant phytohormone JA which is critical for inducing plant defense response [65], and C18:1 is a positive regulator of the jasmonate signaling pathway [66]. Therefore, the decrease in C18:3n3 content in *R. pseudoacacia leaves* in response to *O. robiniae* infection may be connected with synthesis of JA in leaves with galls. This research suggests that the levels of fatty acids in plants are positively correlated with plant defense responses [67]. Opposite results were obtained from another study, indicating that insect elicitor (IE)-induced signaling processes increased free fatty acid levels in maize seedlings, as well as in other plants, and increased fatty acid levels that affected JA accumulation [68]. Studies on castor bean *Ricinus communis* (Euphorbiaceae) suggested that free linolenic acid (C18:2) and linoleic acids (C18:3) increased the most among the free fatty acids in wounded castor bean leaves. Moreover, mechanical wounding activated phospholipase D, a phospholipid-hydrolysing enzyme, leading to diacylglycerol and free C18:3 accumulation [69]. The levels of free C18:2 and C18:3 increased within 1 hour after mechanical wounding of tomato (*Lycopersicon esculentum* var. *Castelmart*) leaves [9]. In plants, unsaturated fatty acids, especially C18 are utilized as raw material to produce numerous aliphatic compounds, including membrane glycerolipids, TAG, cutin/suberin, jasmonates, and nitroalkenes [7]. Another study showed that fatty acids regulate the colonization of jack pine (*Pinus banksiana*) by the invasive mountain pine beetle (*Dendroctonus ponderosae*) and its symbiotic fungus (*Grosmannia clavigera*) [70]. The obtained results suggest that plant tissues (hosts and non-host) enriched with synthetic fatty acids at concentrations typical for jack pine cells were compatible with beetle larvae and the fungus indicating that fatty acids and lipids play a key role in plant-insect interactions defining host suitability to invasive organism [70].

In the mature gall the cell wall was yellow-green, which reflects its transformation from an assimilating organ to a storage-protective one (Fig. 1c). Mesophyll cells are transformed to storage parenchyma (nutrient tissue) with numerous amyloplasts (Fig. 4b-d).

Analysis of ROS parameters as hydrogen peroxide accumulation and the activity of antioxidant enzymes such as catalase and glutathione peroxidase showed different patterns of changes. The level of H_2_O_2_ as a signaling molecule that is a marker of oxidative stress condition was checked in the matured gall [71]. Leaflets with higher levels of H_2_O_2_ may be less attractive for *O. robiniae* for eggs oviposition. Catalase that take a part in decomposition of H_2_O_2_ fluctuated in activity during the experiment. The highest content of H_2_O_2_ was observed in GC in opposition to catalase, whose activity was lower than in IC. In leaflets with gall structure the catalase activity was comparable with global control but the level of H_2_O_2_ showed different trends (Fig. 10). The level of catalase activity increased significantly in leaflets after gall isolation. The same trends were observed in closed gall formed on leaves of *Quercus robur* by *Cynips quercusfolii*, *C. longiventris*, *Neuroterus quercusbaccarum* (Hymenoptera: Cynipidae) [71].

GI featured the least catalase activity during experiment and the level of H_2_O_2_ has the same pattern. In GC the level of both was twice higher than in GI samples. The obtained results suggest that H_2_O_2_ is decomposed via a different way. We found that the level of H_2_O_2_ in gall structure was reduced (Fig. 9). Analysis of GPX (enzymatic pathway of H_2_O_2_ decomposition via glutathione) is used especially in gall structures (Fig. 11). The antioxidant enzyme activity in gall structure depends on the role of the antioxidative system. The activity of catalase that detoxified H_2_O_2_ increased in leaflets after gall isolation (Fig. 10) indicating that in gall formation a special mechanism may exist that reduces the level of H_2_O_2_ without catalase pathway – e.g., with GPX, whose activity significantly increases after gall isolation (Fig. 11). GPX may also equalize the redox state in cells due to equal levels of protein thiol and non-protein thiol (Fig. 12). Infection of leaflets with *O. robiniae* caused an increased protein thiol level in IC, whereas the quantity of this molecule was less in the gall structure. More non-thiol protein was observed in global control leaflets, whereas the content of non-thiol proteins was the lowest in leaflets with gall structures (Fig. 12). Phenols play a part in the defense system before an attack of *O. robiniae*. Therefore, in GC treatments the level of bound and free phenols was the highest. Similar results were obtained from *Triticum aestivum* infected by *Sitobion avenae*, *Sitobion miscanthi*, *Rhopalosiphum padi* and *Rhopalosiphum maidis* (Hemiptera) confirming the suggestion that phenols play a crucial role in adaptation capacity to biotic stress [72]. On the other hand, our observation indicated that the level of phenols is the lowest in the gall structure (Fig. 13). High contents of both free and bound phenols may act as a first line of defense for plants against pathogen attack in global control leaflets with leaves without any sign of infection (Fig. 13). Free phenols may be treated as markers of stress conditions, whereas bounded phenols reduced the level of H_2_O_2_. Phenolic radicals can be reduced by ascorbic reductase, ascorbic acid and peroxidases – components of the antioxidative system. Low levels of H_2_O_2_ in gall structure or leaflet with gall maybe an element of strategy of *O. robiniae* leading to improvement of food quality with reduced accumulation of toxic secondary metabolites [73, 74]*.*

The results obtained from *R. pseudoacacia* indicate a higher level of tannins in leaflets with gall structure in comparison with control plants (Fig. 14). As a defense mechanism, plants use several components such as elicitors that are produced by the decomposition of cell walls [75]. But at the first sight, the detection of a gall inducer may be inhibited. In black locust the elicitor should be recognized at the initial phase to properly engage defense systems [76]*. O. robiniae* larvae probably used a mechanism that did not provoke the defense system.

Reduction in chlorophyll level may occurred due to the inhibition of their biosynthesis or stimulation of their breakdown. This assumption is confirmed by our results indicating significantly lower concentrations of chlorophyllides, which are precursors in the chlorophyll biosynthesis pathway. Similar plant response was observed during foliar gall formation by a psyllid insect *Trioza pallida* (Hemiptera) in *Mallotus philippensis* leaves where total chlorophyll level decreased, whereas the carotenoid content increased in galled leaves [77]. The inhibition in chlorophyll accumulation in response to *O. robiniae* attack may be also a consequence of chloroplast membrane peroxidation induced by ROS, whose production is triggered during gall formation. Lower chlorophyll content in the galled leaf could be also caused by the loss of palisade tissues, chloroplast disintegration, and changes in spongy mesophyll tissue after the leaf is colonized by insects [78]. Reduction in photosynthesis caused by an invasive painted bug *Bagrada hilaris* (Hemiptera) on its host *Brassica oleracea var. botrytis* was observed recently by Guarino et al. [79]. Similar results were obtained by Skuhravá et al. [32], indicating that the galls are green at the beginning of larval development, but when the larvae reach the 3rd instar and begin to pupate they turn yellowish or pink and then dark brown. On the other hand, higher chlorophyll content, greater abundance, and a significant difference in the composition of phytophagous insect species were recorded in flowering trees of *Poincianella pyramidalis* (Fabaceae) in comparison with non-flowering trees [80]. The results suggested that higher nutritional quality of leaf tissue can be more attractive for insects, leading to more differences in species diversity and an abundance of phytophagous insects. On the other hand, average chlorophyll content of unharmed holm oaks (*Quercus suber* and *Q. ilex*) vs. damaged trees by beetle *Coraebus florentinus* (Coleoptera) or by species belonging to beetle *Cerambyx* group did not change among these tree groups [81].

The reduction in monosaccharide content in *R. pseudoacacia* leaves during gall formation (Table 1) may be due to the enhancement in the degradation of photosynthetic pigments contributing to lower efficiency of the photosynthesis process and sugar accumulation. Increasing evidence suggests that insect attack modifies the patterns of carbon allocation in plants. In some studies, it has been reported that remobilization of carbon from damaged and undamaged tissues to stems and roots actual is simulated by herbivory insects [82]. Similarly in *Nicotiana attenuate* (Solanaceae) the levels of soluble sugars (e.g. glucose, fructose, and sucrose) have been shown to remain unchanged in the roots but decreased rapidly in the leaves in response to *Manduca sexta* (Lepidoptera) attack [83]. Sugars may be used as feed for gall inducing insects. The nutritional requirements of the sweet potato whitefly *Bemisia tabaci* a polyphagous homopteran, are provided by the plant phloem on which they feed. Phloem is characterized by high carbohydrate content which satisfies the energy needs of the insect [84]. Moreover, insects prefer host plants with moderate amounts of sugars. High concentrations of sugars are avoided by leafhoppers (Cicadellidae), grasshoppers (Caelifera), and the European corn borer (Crambidae) [73]. The decrease in monosaccharide content in *R. pseudoacacia* leaves also suggests another explanation that low level of monosaccharides may probably promote gall development because some sugars and sugar-alcohol combinations (glucoside and mannoside) delay utilization of other sugars, and they can be harmful for insects [84]. On the other hand, the reduction in monosaccharide content in leaves with galls suggests that these sugars can be utilized to biosynthesize starch, whose level increased after insect attack.

At some stage of development in mature galls, we observed a reduced amount of starch in the nutrient tissue (Fig. 5b-c). We detected partially dissolved cell walls in cells of nutritive tissue with a higher content of fatty acids (Figs. 5d-f). Around the conductive bundles, sclereids were shown to belong to the bundle sheath (Fig. 5a). The stiffness of the galls is provided by the sclerenchyma (woody strengthening tissue), which prevents them from collapsing and protects the larvae feeding inside. This stiffness werent notice in control leaves (Fig. 7a) and in young developing galls. They form a brachysklereids by the lignification of the parenchyma cells (Fig. 7b-f), along the bundles. This is probably the mechanism that protects insect larva from mechanical injury as in the case of the midrib gall moth on *C. langsdorffii* [56].

The increased levels of carotenes in *R. pseudoacacia* leaves with aging galls may be a key mechanism involved in the counteraction of the harmful effect of free radicals generated under biotic stress (Table 1). Carotenes are localized very close to the primary site of ^1^O_2_ production in chloroplasts (i.e. the PSII reaction centre). These pigments protect chlorophyll against its oxidation through quenching or scavenging the free radicals and reduce the damage of the plant cell [85]. However, our results indicate that the increased levels of both *α*- and *β*-carotene in *R. pseudoacacia* leaves attacked by *O. robiniae* may be due to their resistance to oxidation or breakdown under stress conditions, because they show relative biochemically stability in the pigments [86].

Among carotenoids, the contents of xanthophylls such as astaxanthin, neoxanthin, cryptoxanthin, lutein, violaxanthin, and zeaxanthin were higher in leaves with galls, especially in aging gall leaves (Table 1). Xanthophyll pigments protect the photosynthetic apparatus against oxidative stress generated by insect attack, eliminating free radicals with great efficiency [87, 88]. Thus, the accelerated accumulation of xanthophylls observed in late stage of gall formation in *R. pseudoacacia* leaves might be a specific plant reaction to the oxidative stress as evidenced by the increase in the H_2_O_2_ level. On the other hand, earlier work showed that antioxidants such as ascorbic acid, total carotenoids, non-protein thiols, and catalase decreased in plants in response to pathogen attack [89]. The degradation of carotenoids, chlorophylls, and the production of volatile organic compounds may be symptoms of autotoxicity or a result of strategic management of ROS by plants during biotic stress [87]. Moreover, biochemical evidence indicated that violaxanthin, which is representative of xanthophylls, also acts as a precursor of ABA – a key phytohormone involved in plant adaptation to biotic stress [90, 91]. To summarize, the increased carotenoid content triggered by the gall in *R. pseudoacacia* leaves may protect the thylakoid membrane from ROS-mediated oxidative damage and thereby prevent disturbances in the photosynthetic apparatus.

## Data Availability

All data generated or analysed during this study are included in this published article. The datasets generated and/or analysed during the current study are available from the corresponding author on reasonable request.
